# An Empirical Analysis on the Longevity of Dairy Cows in Relation to Economic Herd Performance

**DOI:** 10.3389/fvets.2021.646672

**Published:** 2021-04-12

**Authors:** Imke Vredenberg, Ruozhu Han, Monique Mourits, Henk Hogeveen, Wilma Steeneveld

**Affiliations:** ^1^Department of Population Health Sciences, Faculty of Veterinary Medicine, Utrecht University, Utrecht, Netherlands; ^2^Business Economics Group, Wageningen University, Wageningen, Netherlands

**Keywords:** dairy (cows), economics, longevity, culling age, lifetime milk production, accounting data

## Abstract

Several studies have stated the various effects of an increased dairy cow longevity on economic herd performance, but empirical studies are lacking. This study aimed to investigate the association between longevity of dairy cows and the economic performance of dairy herds based on longitudinal Dutch accounting data. Herd and farm accounting data (*n* = 855 herds) over the years 2007–2016 were analyzed. Herd data contained yearly averages on longevity features, herd size and several production variables. Longevity was defined as the age of cows at culling and by lifetime milk production of culled cows. Farm accounting data contained yearly averages on revenues, fixed and variable costs of the herds, by which gross margins were defined. Data was analyzed using generalized linear mixed modeling, with gross margin as dependent variable. The independent variables consisted of average age of culled cows, average lifetime production of culled cows, year, herd size, herd intensity (milk production per ha), herd expansion rate, soil type, milking system, successor availability, total full-time equivalent, heifer ratio (% of heifers per cow) and use of outsourced heifer rearing. Herd was included as a random effect to account for the heterogeneity among herds. Descriptive statistics showed that the average age of culled cows was 5.87 (STD = 0.78) years and the average lifetime milk production of culled cows was 31.87 (STD = 7.56) tons per cow with an average herd size of 89 cows (STD = 38.85). The average age of culled cows was stable over the 10 years (variation between 5.79 AND 5.90 years). The gross margin was on average €24.80/100 kg milk (STD = 4.67), with the lowest value in year 2009 and the highest value in year 2013. Gross margin was not significantly associated with age of culled cows and lifetime milk production of culled cows. Variance in longevity between herds was large (STD = 0.78 years) but herds with a higher longevity did not perform economically better nor worse than herds resulting in lower longevity. This indicates that, within current practice, there is potential for improving longevity in order to meet society's concerns on animal welfare and environmental pollution, without affecting the economic performance of the herd.

## Introduction

Longevity of a dairy cow can be defined as the total lifespan of a cow or as the length of productive life (1). The productive lifespan of average dairy cows in industrialized countries varies from <3 years (2) to at least 4.5 year (3). These cows calve for the first time at ~2 years of age, which brings their total lifespan from birth to departure from the herd between 4.5 and 6.5 years. The average total lifespan of dairy cows in the Netherlands in 2018 was 5.5 years (4), while the natural lifespan of dairy cattle is ~20 years (5). Hence, cows are culled well before the end of their natural lifespan, which is common for animals in dairy livestock production. The decision to cull a cow is primarily driven by economic considerations as made by the farmer. Therefore, dairy replacement management decisions largely determine the average productive lifespan of dairy cattle (6). Decisions to cull and replace a dairy cow are driven by the cow's level of production, reproduction and health in comparison to the other cows in the herd and the available replacement animals. In the Netherlands, the main culling reasons in 2011 were poor fertility, mastitis and claw disorders (7).

When cows have a prolonged longevity less replacement is needed, and therefore total rearing costs will be lower and rearing costs are spread out over a longer productive life. In the Netherlands, rearing costs of a heifer are on average between €1,423 and 1,715 per heifer (8), reflecting one of the highest dairy production costs. Moreover, a higher longevity will result in more cows in higher parities, and thus in a higher proportion of cows in higher producing age groups, and thus a higher average milk production of the herd. Under milk quota circumstances a higher herd production does have little value, but the farmer then has the option to reduce the herd size due to a higher milk production per cow. A higher longevity might, however, also result in disadvantages, such as increased health and reproduction problems and a reduction in genetic improvement (9).

Besides economic consequences, an increase in longevity will also have environmental and social consequences. Cows with an increased longevity produce less methane per kg of milk (10), improve environmental sustainability (11) and indicate good animal welfare on the farm (12). Impacts on the environment and animal welfare have become increasingly important in public debate.

As stated in several studies [e.g., (1, 13)] a higher longevity can result in less rearing costs and increased returns from a higher lifetime milk production. Empirical studies that support these expectations are, however, lacking. So, it is not yet known from practice, whether farms with a higher longevity perform economically better than farms with a lower longevity of the cows.

The aim of this research is to investigate the association between longevity of dairy cows and the economic performance of dairy herds based on available Dutch accounting data.

## Materials and Methods

### Data

Anonymized yearly herd level data was obtained from a Dutch accounting agency (Flynth, Arnhem, the Netherlands). The data represented 2,809 herds with 30,170 yearly records from 2007–2016. The accounting dataset contained information on economic performance indicated by revenues (e.g., milk revenues) and fixed and variable costs (e.g., feed costs and veterinary costs), as well as on general herd characteristics (e.g., soil type, number of full-time employees). Economic data was expressed in absolute values and in ratios per 100 kg milk produced per year.

The annual farm accountancy data of these 2,809 herds were subsequently merged with herd performance data derived from the Cattle Improvement Cooperative (CRV, Arnhem, the Netherlands). These data included herd information on herd size, longevity features (e.g., age of the cows in days and number of production days of the cows) and production, such as 305-day milk production and 305-day percentage fat and protein. For 2,105 herds CRV herd performance data was available.

### Data Management

**Figure 1:**
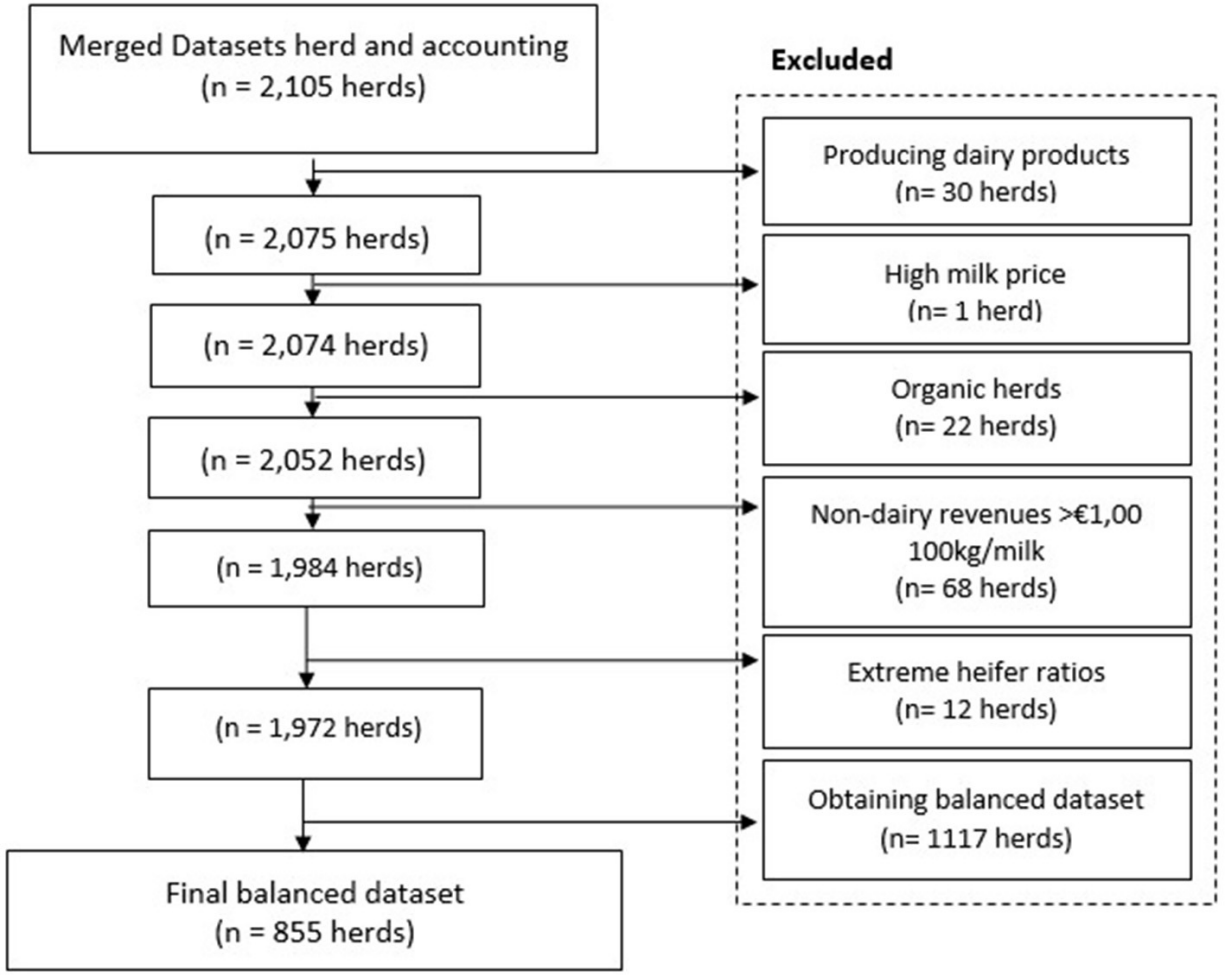
Data editing steps, starting with the merged dataset till the final dataset containing 855 herds.

Only data from commercial dairy herds were selected for further analysis. A commercial Dutch dairy farm was defined as a herd with more than 30 cows and an average 305-day milk production above 4,000 kg per cow. It was argued that the amount of labor needed to manage at least 30 cows indicates a commercial way of farming. Moreover, by using 30 cows, non-commercial farms like hobby herds and petting farms were excluded. Furthermore, herds with missing values on important variables (e.g., 305-day milk production, age at culling, lifetime milk production of culled cows and number of heifers) were removed (Figure 1). Subsequently, organic herds (*n* = 22 herds) and a herd with an unexplainable high milk revenue (*n* = 1 herd) were excluded, because on all these herds milk revenues were distinct higher than on conventional herds. Also, herds producing dairy products (e.g., cheese, yogurt) (*n* = 30 herds), with non-dairy revenues higher than €1.00/100 kg milk (*n* = 68 herds) or with an extreme heifer ratio (≤0.08; ≥0.5) (*n* = 12 herds) were excluded. Heifer ratio was calculated by the number of heifers that have calved divided by the average number of milking cows annually. It was argued that these herds may had other business activities than only dairy production, like cow trading, crop production, or running a farm shop. Since the longevity performance of herds can be better analyzed based on data of several years only farms with continuous data of 10 years were selected. As a consequence, farms that quitted farming or changed accounting agency during the evaluated period were excluded from further analysis. The final balanced dataset contained information on 855 commercial dairy herds with 10 years of consecutive observations (Figure 1).

**Figure 2:**
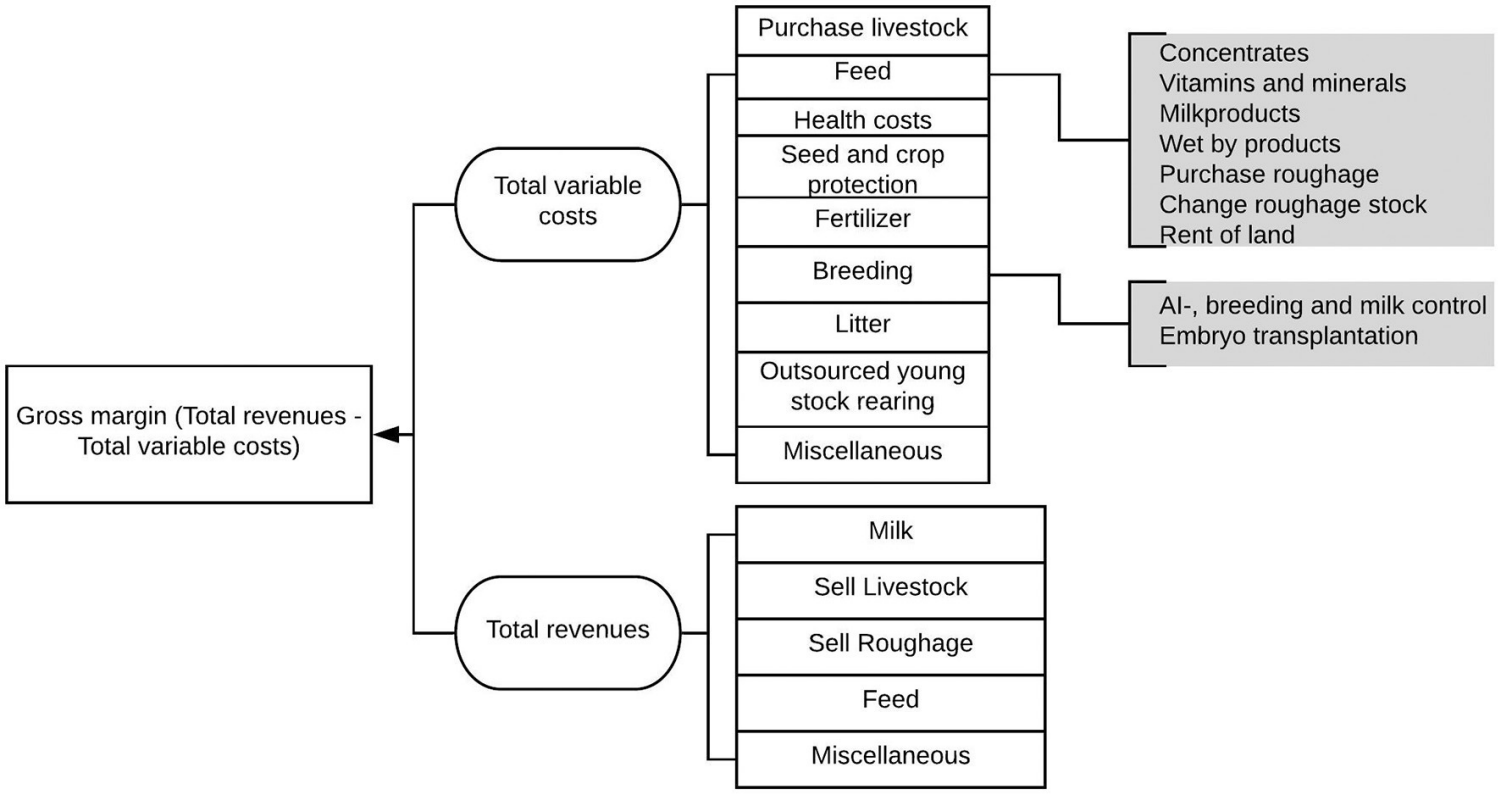
Overview of revenues and costs accounted for in the gross margin of the herds for dairy production. Examples of miscellaneous costs are costs related to water, electricity and manure disposal. Examples of miscellaneous revenues are subsidies and rental of barn space. Feed revenues include, for instance, the sales of silage.

The average age and lifetime milk production of culled cows were chosen to reflect the longevity features of the herd. Other selected variables in the data were selected based on an expected association with gross margin. The selected variables were herd size, use of outsourced young stock rearing (yes/no), number of full-time employees, land area, whether the farmer has a successor (yes/no), soil type (sand vs. non-sand), milking system (conventional vs. automatic milking system), total herd milk production and number of cows per ha. Soil type was selected as Dutch farms producing on different soil types (especially clay vs. sand) differ in milk revenues and costs for purchasing feed (14). The variable having a successor was selected as it was expected that farmers with a successor make different management decisions than those without a successor, hence, resulting in different gross margins. In addition, the variables herd expansion, production intensity and heifer ratio were calculated. Herd expansion reflected the ratio of herd size changes on the basis of reference year 2007. Production intensity indicated the annual average milk production in tons per hectare. To analyse the economic performance of herds, the gross margin for dairy production was calculated as the total revenues minus the total variable costs and was expressed in euros per 100 kg milk produced (Figure 2).

### Data Analysis

The linearity of the relationships between the selected variables and gross margin were visually inspected by creating boxplots. In order to avoid multicollinearity, a Pearson correlation coefficient above 0.6 between continuous independent variables was used to remove the strongly correlated variables. Consequently, the total ha of the farm (highly correlated with herd size) and the average number of cows per ha (highly correlated with average tons of milk production per ha) were removed from further analysis. Two generalized linear mixed models (GLMM) were developed to analyse the association of dairy cow longevity (measured either by age or by lifetime milk production of culled cow) with economic performance of herds. The dependent variable of these models was the gross margin of the herd, reflecting the economic performance. The independent variables consisted of age or lifetime milk production of culled cows (hence 2 models) in combination with the independent variables soil type, milking system, whether a successor was available, whether young stock was outsourced, number of full-time employee, heifer ratio, herd expansion, herd size and herd intensity. A year variable was forced into both models to account for potential year effects (e.g., milk price changes). Moreover, to capture the unobserved herd related heterogeneity, such as management strategy, a herd variable was entered into the models as a random effect. To account for the covariance among the consecutive gross margin measurements within herds, competing covariance structures (i.e., independent, compound symmetry, first-order autoregressive, first-order autoregressive moving average and unstructured) were tested for their fit. Based on the Akaike information criterion, the unstructured covariance structure resulted in the best model fit and was eventually used in the presented models.

## Results

### Descriptive Statistics

Over the evaluated period of 2007 to 2016, the average age of culled cows was equal to 5.87 years. Meanwhile, the average lifetime milk production of culled cows was 31.87 tons per cow. The standard deviations (STD) of the longevity variables between farms, were larger than the average STD within farms. The STD of age of culled cows between farms was 0.78 years, while the average STD within farms was equal to 0.59 years. For the lifetime milk production, the STD between farms was 7.56 tons, while the average STD within farms was 5.37 tons. The average herd size over the evaluated period was almost 89 cows (Table 1) and increased from, on average, 76 cows in 2007 to, on average, 103 cows in 2016.

**Table 1:** Descriptive statistics on continuous variables over herds and years (*n* = 855 herds).

	**Description (unit)**	**Mean**	**STD**	**5% percentile**	**95% percentile**
Age culled cows	Age of culled cows (years)	5.87	0.78	4.75	7.24
Lifetime milk production	Lifetime milk production of culled cows (tons)	31.87	7.56	20.73	45.16
Total FTE	Total number of full-time employees	1.88	0.72	1	3
Heifer ratio	Number of calved heifers per average cow present in the herd	0.24	0.06	0.15	0.33
Herd size	Number of cows present in the herd	88.87	38.85	44	161
Herd expansion	Herd size change from 2007 to 2016 in relation to base year 2007	1.15	0.23	0.92	1.57
Herd intensity	Milk production per ha (tons)	15.84	4.46	9.97	23.63

Average total variable costs were €14.54/100 kg milk, while the average total revenues equalled €39.34/100 kg milk. The average gross margin over the evaluated period was €24.8/100 kg milk (Table 2).

**Table 2:** Descriptive statistics on variable costs, revenues and gross margin (in €/100 kg milk) over herds and years (*n* = 855 herds).

		**Mean**	**STD**
Variable costs	Feed	8.95	2.43
	Purchase livestock	0.47	1.47
	Fertilizer	1.04	0.37
	Seed and crop protection	0.56	0.30
	Health	0.98	0.41
	Breeding	0.95	0.31
	Outsourced young stock rearing	0.17	0.67
	Litter	0.46	0.35
	Miscellaneous^a^	0.96	0.38
	Total	14.54	1.99
Revenues	Milk	36.24	4.70
	Sell livestock	2.96	1.65
	Sell roughage	0.13	0.43
	Feed^b^	0.0001	0.01
	Miscellaneous^c^	0.01	0.05
	Total	39.34	5.15
Gross margin	Total revenues – total variable costs	24.80	4.67

a *e.g., water, electricity and manure disposal*.

b *Selling of silage*.

c *e.g., subsidies and rental of barn space*.

The descriptive statistics on age of culled cows, lifetime milk production of culled cows, and gross margin for different categories of the categorical variables year, soil type, milking system, having a successor and making use of outsourced youngstock rearing are presented in Table 3. The average age of culled cows was rather constant over the years (variation between 5.79 and 5.90 years). A slight increase in average lifetime milk production of culled cows was displayed throughout the evaluated period (30.82–32.65 tons). Among the categorical variables, such as soil type, milking system, whether having a successor and whether young stock rearing was outsourced, there were almost no differences in average age of culled cows and average lifetime milk production of culled cows. The average gross margin varied substantial between years, with the lowest value realized in 2009 (€18.48/100 kg milk), and the highest value in 2013 (€29.90/100 kg milk). The average gross margin tended to be higher in farms with sandy soil and farms with a conventional milking system, compared to farms with non-sandy soil and an automatic milking system, respectively. In addition, herds outsourcing their young stock rearing had a lower average gross margin (€22.92/100 kg milk) than herds not outsourcing young stock rearing (€25.02/100 kg milk).

**Table 3:** The number of observations, mean and standard deviation (STD) of average longevity variables (age and lifetime milk production of culled cows) and gross margin per categorical variable.

			**Age of culled cows (year)**		**Lifetime milk production of culled cows (tons)**		**Gross margin (**€**/100 kg milk)**	
		***N* obs**	**Mean**	**STD**	**Mean**	**STD**	**Mean**	**STD**
Year^a^	2007		5.87	0.84	30.82	7.72	26.58	2.56
	2008		5.94	0.83	31.74	7.80	25.13	3.06
	2009		5.89	0.76	31.59	7.11	18.48	2.65
	2010		5.86	0.75	31.74	7.49	25.20	2.59
	2011		5.79	0.77	31.43	7.68	28.00	2.87
	2012		5.78	0.73	31.60	7.39	24.98	2.96
	2013		5.90	0.84	32.48	7.97	29.29	3.14
	2014		5.89	0.74	32.45	7.32	29.09	3.38
	2015		5.86	0.78	32.24	7.55	21.28	3.47
	2016		5.89	0.73	32.65	7.32	20.01	3.26
Soil type	Sandy soil	6,067	5.86	0.79	31.77	7.51	24.95	4.67
	Other soil	2,483	5.88	0.76	32.14	7.65	24.44	4.64
Milking system	Conventional	7,023	5.89	0.79	31.91	7.70	24.90	4.61
	Automatic	1,527	5.74	0.70	31.71	6.88	24.37	4.92
Successor	No	5,410	5.87	0.79	31.66	7.43	24.84	4.69
	Yes	3,140	5.85	0.77	32.24	7.75	24.74	4.64
Outsourcing young	No	7,674	5.86	0.78	31.73	7.56	25.02	4.62
stock rearing	Yes	876	5.90	0.75	33.16	7.44	22.92	4.67

a *In comparison to the other categorical variables, each year category consists of only one herd measurement*.

### Regression Analysis

Table 4 presents the results of the developed GLMM to study the association between longevity (age of culled cows and lifetime milk production of culled cows) and economic herd performance (gross margin). Overall, the results did not demonstrate any significant association between the longevity variables and gross margin. Of the evaluated independent variables soil type, milking system, use of outsourced heifer rearing, heifer ratio and herd intensity were significantly associated with gross margin. The strength of these associations was comparable among the two models. The use of outsourced youngstock rearing was associated with on average a €1.02/100 kg milk lower gross margins compared to the use of only own youngstock rearing. In addition, herds on sandy soils were associated with a €0.56/100 kg milk higher gross margins than herds on non-sandy soils, while the use of an automatic milking system was associated with €0.52/100 kg milk lower gross margins than on farms with a conventional milking system. One ton of milk production increase per ha was associated with an decrease in gross margin by €0.13/100 kg milk. An increase in heifer ratio of 0.1 (hence, having 10% more calved heifers in relation to milking cows) was associated with an increase in gross margin by €0.08/100 kg milk.

**Table 4:** Results of the generalized linear mixed models on association between longevity (age of culled cows and lifetime milk production of culled cows) and gross margin (in €/100 kg milk).

		**Age of culled cows**		**Lifetime milk production of culled cows**	
		**Estimate**	***P*-value**	**Estimate**	***P*-value**
Intercept		28.690	<0.0001	28.770	<0.0001
Year	2007	Ref.^a^		Ref.^a^	
	2008	−1.356	<0.0001	−1.352	<0.0001
	2009	−7.959	<0.0001	−7.955	<0.0001
	2010	−1.196	<0.0001	−1.190	<0.0001
	2011	1.640	<0.0001	1.645	<0.0001
	2012	−1.386	<0.0001	−1.380	<0.0001
	2013	3.044	<0.0001	3.054	<0.0001
	2014	2.881	<0.0001	2.891	<0.0001
	2015	−4.825	<0.0001	−4.816	<0.0001
	2016	−5.989	<0.0001	−5.978	<0.0001
Age culled cows (years)		−0.017	0.5920		
Lifetime milk production (tons)				−0.006	0.0915
Soil type	Sandy soil	Ref.^a^		Ref.^a^	
	Other soil	−0.564	0.0004	−0.561	0.0004
Milking system	Conventional	Ref.^a^		Ref.^a^	
	Automatic	−0.519	<0.0001	−0.518	<0.0001
Successor	No	Ref.^a^		Ref.^a^	
	Yes	−0.070	0.4165	−0.068	0.4302
Outsourcing young stock rearing	No	Ref.^a^		Ref.^a^	
	Yes	−1.023	<0.0001	−1.020	<0.0001
Total full-time employee		−0.025	0.6860	−0.024	0.7064
Heifer ratio		0.823	0.0241	0.805	0.0273
Herd expansion		−0.041	0.8614	−0.040	0.8631
Herd size		0.001	0.7666	0.001	0.7997
Herd intensity (tons milk/ha)		−0.129	<0.0001	−0.128	<0.0001

a *This category is used as reference category in the regression analysis*.

The marginal *R*^2^ (variance explained by fixed effects) and the conditional *R*^2^ (variance explained by entire model) of the model on age of culled cows were 0.60 and 0.80, respectively. The same values were found for the model on lifetime milk production of culled cows.

## Discussion

The average age of culled cows was rather constant over the evaluated period (variation between 5.79 and 5.90 years). Corresponding averaged STD of 0.78 years, however, indicated distinct differences in culling age between herds. Similarly, averaged observed variance in lifetime milk production (STD 7.56 tons) indicated relevant differences between herds, while the average annual lifetime milk production of culled cows only slightly varied around a value of 31.9 tons of milk. Hence on herd population level, longevity did not alter much during the evaluated years 2007–2016. The gross margin was on average €24.80/100 kg milk (STD = 4.67). It might be possible that a very small proportion of this gross margin was due to non-dairy production. This will, however, be a neglectable small proportion as dairy herds with distinct other business activities were excluded.

Modeling results indicated that longevity (age and lifetime milk production of culled cows) was not significantly associated with the gross margin of commercial Dutch dairy herds. Herds with higher longevity did not have a significantly higher nor lower gross margin than herds with a lower longevity. Although it is frequently reported that a higher longevity will have positive economic consequences because of less young stock rearing and a higher average milk production [e.g., (1, 13)], this was not observed in the observational data used in the current study. Negative effects of a higher longevity, like the reduction in livestock sales due to a reduction in the removal of dairy cows or increased health and/or reproduction costs (15, 16), might have leveled out potential positive consequences. Moreover, this balance between positive and negative effects between years might have been influenced by differences in price levels as well as by management changes triggered by policy alterations (e.g., abolishment milk quota). The effects of longevity on specific costs or revenues (e.g., health costs, livestock sales) can be investigated in the future.

The independent variable year was strongly associated with the gross margin, which was largely caused by the differences in milk price between the years. Since the milk price in the Netherlands was lowest in 2009 and highest in 2013 (respectively, €27.51/100 kg milk and €43.04/100 kg milk) (17), it was to be expected that the year 2009 was associated with the lowest gross margin, and the year 2013 with the highest gross margin (Table 3). Moreover, the years 2013–2015 (period in which farmers already anticipated on the abolishment of the milk quota system in 2015) can be considered as years where farmers might have made different strategic management decisions (e.g., building new barns, rearing more or less youngstock, and culling more or less cows) than in the more stable (quota restricted) years before that period, resulting in some year specific influences. To account for any specific effects between longevity and year that might have affected the gross margin, interaction terms have been tested but these turned out to be insignificant (data not shown).

It remains, however, inherent to field data that results are influenced by external changes, such as national agricultural policies and changes in price levels. Moreover, the gross margin is only a partial measure of farm profitability. Farm assets such as the modernity of the farm buildings and farm machinery, the quality and amount of land and the amount of own labor. Hence, the fixed costs are not taken into account. It is difficult to work with economic measures such as net profit because in accountancy data, the value of these assets is not well-known. In the future, other methods, such as the use of an efficiency analysis (18, 19), where the farm's relative efficiency in terms of producing milk given a certain amount of resources is evaluated may provide a more complete economic view of the association between cow longevity and farm performance. On the other hand, because most of the fixed costs are linked to farm structure which cannot be changed in the short run, gross margin does provide a good indication of the short term profitability of a farm.

The independent variables milking system, use of outsourced heifer rearing, herd intensity, soil type and heifer ratio were not significantly associated with the gross margin (Table 4). Herds with an automatic milking system had on average a lower economic performance than herds with a conventional milking system, which was an expected association based on earlier findings of Bijl et al. (20) and Steeneveld et al. (19). Making use of outsourced young stock rearing was also associated with a lower gross margin than the use of own young stock rearing. This was expected as outsourced young stock rearing means that all costs (feed, housing and labor) are represented as a variable costs in the gross margin. While with own young stock rearing, only the feed costs [approximately one-third of the total costs of young stock rearing; (8)] are represented in the variable costs and housing and labor are fixed costs. More intensive farms (defined as more kg milk per hectare) were associated with a lower gross margin, most probably due to higher purchasing feed costs than less intensive farms. Also farms on non-sandy soil were associated with a lower gross margin due to lower milk revenues than on sandy soil (data not shown). Heifer ratio was positively associated with gross margin, indicating that farms that had more calved heifers per milking cow had a higher gross margin in comparison with farms that have less calved heifers per milking cow. This was to some extent an unexpected association as generally the amount of young stock is reflected in the heifer ratio. A higher heifer ratio, hence more young stock, would, in theory, lead to more variable costs and hence a lower gross margin. This assumption is, however, only valid in a stable farm production system, which was not the case during the evaluated period. Triggered by the abolishment of the milk quota in 2015, farmers already anticipated in the preceding years 2013–2014 by increasing their young stock rearing resulting in higher rearing costs, while the revenues resulting from this accelerated heifer rearing were not obtained until 2 years later. Due to this rearing time lag the increase in youngstock rearing was not direct captured by the heifer ratio. Hence, increased rearing costs were related to unaltered heifer ratios, while the additional revenues as a result of the increased rearing were related to higher ratios.

Longevity of dairy cows has been mostly evaluated in terms of culling of individual cows, as longevity is determined by the moment of the cows' departure from the herd for voluntary or involuntary reasons. Culling reasons and risk factors for culling are intensively studied worldwide [e.g., (21–23)]. Also studies on optimization of culling decisions and costs of culling (24–26) are performed. Empirical analysis on the economic consequences of a higher longevity or a lower culling rate are however lacking. Only De Vries (6) and De Vries and Mercondes (13) discussed the economic consequences of a higher longevity at the herd level and stressed lower replacement costs and a higher lifetime milk production. It was, however, also mentioned that a higher longevity is not necessarily profitable per cow per year, since the facilities are the most limiting factor (13). Our study is the first study that analyzed the economic consequences of longevity in an empirical way, and the Dutch commercial farm economics was taken into account by using farm accounting data. The gross margin was expressed per 100 kg milk per year as under Dutch milk quota circumstances (until 2015) kg of milk was the most limiting factor.

De Vries and Mercondes (13) argued that it is conceivable that society will start to demand a higher longevity that is more in line with the natural life expectancy, given that health problems are major drivers of culling at a young age. According to the (27) an increase in longevity of 2 years would be desirable. However, forcefully increasing longevity to such an extent, without adjustments on health management will, however, have negative effects, such as increasing incidences of diseases. Therefore, additional costs for changes in health management and housing (access to pasture, improving cow comfort) will be needed to improve longevity in a structural way (13). Although observational studies, due to a lack of experimental control, have disadvantages in interpretation, the data in this study may help the dairy sector in their decisions regarding in setting their ambitions regarding longevity.

In conclusion, longevity (age at culling, lifetime milk production of culled cows) was not statistical significantly associated with the gross margin of Dutch dairy herds, based on observational longevity and accounting data. Variance in longevity between herds was large but results demonstrated that herds with a higher longevity did not perform economically better nor worse than herds resulting in lower longevity. This indicates that within current practice there is potential for improving longevity in order to meet society's concerns on animal welfare and environmental pollution without affecting the economic performance of the herd.

## Data Availability Statement

The raw data supporting the conclusions of this article will be made available by the authors, without undue reservation.

## Author Contributions

IV and RH: data analysis and drafting the manuscript. MM and WS: drafting the manuscript and critical revision of the article. HH: critical revision of the article. All authors contributed to the article and approved the submitted version.

## Conflict of Interest

The authors declare that the research was conducted in the absence of any commercial or financial relationships that could be construed as a potential conflict of interest.
